# Working well: strategies to strengthen the workforce of the Indigenous primary healthcare sector

**DOI:** 10.1186/s12913-019-4750-5

**Published:** 2019-11-29

**Authors:** Crystal Jongen, Janya McCalman, Sandy Campbell, Ruth Fagan

**Affiliations:** 1 Centre for Indigenous Health Equity Research, Central Queensland University, Cairns, Queensland Australia; 2 Gurriny Yealamucka Health Service, Yarrabah, Australia

**Keywords:** Indigenous, Aboriginal, Workforce, Primary health care, Community control

## Abstract

**Background:**

The capacity of the Indigenous primary healthcare (PHC) sector to continue playing a crucial role in meeting the health needs of Aboriginal and Torres Strait Islander Australians is in large part reliant on the skills, motivation and experience of its workforce. While exhibiting many workforce strengths, the sector faces significant challenges in building and maintaining a strong and stable workforce. Drawing on data from one Aboriginal Community Controlled Health Service (ACCHS), this study reports what is working well and what could be improved to strengthen the Indigenous PHC sector workforce.

**Methods:**

Using grounded theory methods, interviews with 17 ACCHS staff from a range of organisational positions were transcribed, coded and analysed. This paper focuses on the strategies identified that contribute towards strengthening the Indigenous PHC workforce.

**Results:**

Four overarching strategies for Indigenous PHC workforce strengthening were identified. These were *Strengthening Workforce Stability, Having Strong Leadership, Growing Capacity,* and *Working Well Together*. A range of enabling factors at the macro, community, organisational and individual levels were also identified.

**Conclusion:**

Indigenous PHC services are already implementing many important workforce-development strategies that are having a positive impact on the sector. There are also several persistent challenges which need to be addressed through action at organisational and structural levels. Approaches to workforce strengthening in Indigenous PHC should be tailored to local needs to ensure they address the unique workforce challenges experienced in different contexts.

## Introduction

Aboriginal and Torres Strait Islander (hereafter respectfully referred to as Indigenous) peoples in Australia experience many inequities in health outcomes which could be addressed by better access to effective and appropriate primary health care services [1]. However, Indigenous people face several barriers to accessing health care, including cost of healthcare, transport and location barriers, language barriers, and barriers due to a lack of cultural safety in many healthcare services [2]. Indigenous primary healthcare (PHC) services play an important role in overcoming these healthcare access barriers through the provision of culturally appropriate comprehensive primary healthcare [3]. However, to be able to meet the healthcare needs of Indigenous peoples, Indigenous PHC services need strong and stable workforces.

Indigenous PHC services depend on their workforce, and international studies have found that efforts to make care more effective, efficient, patient-centered and integrated are usually made or broken by employees [4]. However, the efforts of Indigenous PHC services to provide consistent quality healthcare service delivery to their clients can be impeded by key challenges relating to the health workforce. There are system wide shortages of healthcare professionals, particularly in regional and remote locations (e.g. [5]) with issues related to staffing levels, retention and turnover being identified as some of the top challenges experienced by Indigenous PHC services nationally [6].

The National Aboriginal and Torres Strait Islander Health Workforce Strategic Framework 2016–2023 identified six priority areas for building a strong and supported health workforce. These are: 1) improving recruitment and retention of Aboriginal and Torres Strait Islander health professionals; 2) improving workforce skills and capacity; 3) supporting the sector to provide culturally-safe and responsive workplace environments; 4) increasing the number of Aboriginal and Torres Strait Islander students studying for qualifications in health; 5) improving completion/graduation and employment rates for Aboriginal and Torres Strait Islander health students; and 6) improving information for health workforce planning and policy development [6]. However, many Indigenous PHC services struggle to implement these priorities [7]. A systematic review found that barriers to Indigenous health workforce retention include systemic factors such as limited organisational funding and inadequate remuneration and limited career pathways; organisational factors such as heavy workloads and demands, lack of support from management and lack of mentoring, and professional development opportunities; and proximity to community on the individual level [8]. Factors that affect the engagement of Indigenous people with education, training and employment included racism, family and community responsibilities, stress, isolation and poor secondary education [9].

A range of important strategies to support the sustainability and development of the Indigenous PHC workforce particularly in rural and remote areas have been identified in systematic literature reviews [9–11]. Opportunities to develop professionals’ workforce skills and competencies through training, mentorship, accreditation and promotion are especially important for rural Indigenous PHC workforces. Other critical strategies include: fostering cohesive and harmonious workplaces through strong teamwork, support from colleagues and shared purpose and identity; strong leadership and management which provides effective supervision and supports effective communication across the organisations; and providing realistic and competitive remuneration [9–11].

The engagement and retention of Indigenous health professionals in Indigenous PHC is a particularly important strategy. There is evidence that Indigenous health professionals can help to overcome key cultural and communication barriers for Indigenous people accessing health care [8, 12–14]. For example, there is evidence to suggest that Aboriginal and Torres Strait Islander Health Workers (ATSIHW), otherwise known as Indigenous Health Workers (IHW), may help to improve attendance at appointments and acceptance of treatment and assessment recommendations [9], reduce discharge against medical advice, increase patient contact time, enhance patient referral linkages, and improve patient follow up practices [13]. Preliminary evidence also shows potential for IHWs to improve diabetes screening [15, 16] and care management processes [17, 18], as well as palliative care [19] and maternal and infant care [20].

While there is no nationally consistent definition of an IHW or ATSIHW, generally an IHW is a person who: 1) identifies as being of Aboriginal and/or Torres Strait Islander descent; 2) holds an Aboriginal and Torres Strait Islander Primary Health Care Qualification; and 3) adopts a culturally safe and holistic approach to health care [2]. IHW’s play an essential and unique role in Indigenous PHC services. Their roles encompass a range of comprehensive PHC activities including assessment, intervention, health promotion, disease prevention and chronic disease management, with IHW roles often adapted to local needs and contexts. IHW’s are also play a crucial role in providing culturally safe care to clients through advocating for clients, explaining their cultural needs and educating other healthcare professionals. Indigenous health professionals support non-Indigenous health professionals to provide culturally appropriate care through providing cultural, social and community mentorship and education, acting as cultural brokers, helping to increase patient trust and safety and therefore improving care [8, 9, 12, 13, 20].

However, Indigenous Australians in general are under-represented in the health workforce, and IHWs, in particular, have high turnover rates [8]. While there has been an overall growth in the number of IHWs between 2006 and 2016, this growth has not matched the population growth of Aboriginal and Torres Strait Islander peoples leading to concerns that little is being done to increase recruitment and retention of this workforce [21]. IHW career development pathways have also been limited by significant variation in IHW roles, scopes of practice, education and career pathways [14].

The engagement and retention of Indigenous health professionals has been supported by co-worker support and peer mentorship; inclusiveness and cultural safety in the workplace and culturally competent human resources policy and practice; access to clinical and cultural supervision; clear definition of roles and enhanced role recognition; job security and adequate remuneration; and support for expanded roles and strong career pathways [8, 9, 11]. Career pathways have been enhanced through explicit and appropriate training and education pathways and strategies that allow for a combination of formal education and training as well as cultural guidance and support, or learning through mentoring and work shadowing [8, 9, 11]. For non-Indigenous professionals in Indigenous PHC in particular, additional factors which impact on their longevity and effectiveness in rural Indigenous PHC include cultural competence, clinical experience, qualifications and skills and perceived connection with the local community and Aboriginal colleagues [9].

This paper reports a qualitative exploration of the workforce strengths and challenges of one ACCHS. An ACCHS is defined by the National Aboriginal Community Controlled Health Organisation (NACCHO) as an incorporated Aboriginal organisation, initiated by and based in a local Aboriginal community, governed by an Aboriginal body which is elected by the local Aboriginal community and delivering an holistic and culturally appropriate health service to the Community which controls it (NACCHO, 2012 in [22]). Established as advocacy services in the 1960s and 1970s in response to the poor health of Indigenous people and communities and failure of mainstream services to provide adequate health care, and enabled by government policies and funding, ACCHSs are an essential part of the Indigenous PHC sector and are considered crucial in driving efforts to close the gap in Indigenous health outcomes in Australia [22, 23]. ACCHSs address health inequalities by providing a culturally appropriate alternative to mainstream medical services [24] and reducing healthcare access barriers for Indigenous people [3]. ACCHSs have been pioneers in comprehensive PHC [25] and have demonstrated superior performance to mainstream services on a range of healthcare quality, performance and delivery outcomes [3, 25, 26]. ACCHSs also play an important role in training and developing the health workforce, with approximately 50% of the ACHHS health workforce being Indigenous [3].

The current study was undertaken in Gurriny Yealamucka Health Service (Gurriny), located in Yarrabah, North Queensland. Yarrabah is the largest discrete Aboriginal community in Australia. In 2014, management and accountability for PHC services in Yarrabah were transferred from Queensland Health (QH) to community control through Gurriny. In the 4 years post-transition, Gurriny grew employment of local people by more than 75% to improve culturally safe healthcare to Yarrabah’s 3472 clients, and achieved optimal practitioner to client ratios and workforce stability in some areas [27, 28]. However, multiple funding sources with separate agendas and accountabilities created disjointed workforce planning [29]. Gurriny management considered that further improvements were required in: Indigenous leadership, strengths, career development, wellbeing, competencies, roles/ professions, responsibility, control, accountability, liability, performance/contribution, retention, progression, and underpinning systems and processes. Such issues were the subject of this study. The key research question was: what are the enabling conditions and strategies for a best practice workforce model for one ACCHS. The findings from this study are discussed in the context of broader literature on workforce enhancement for Indigenous PHC and Indigenous health professionals.

## Methods

Using grounded theory methods, we initially conducted interviews with a purposive sample of three information-rich Gurriny staff to get perspectives on what was working well and what could be working better to build a strong workforce. The selection of these participants was based on the length of time these staff had been working at Gurriny and the strength of knowledge, experience and interest in Gurriny’s workforce issues and development. Interviews were conducted by one researcher (SC) and audio recorded. Recorded interviews were de-identified, transcribed by a professional transcriber, and imported into the qualitative data analysis software NVIVO, where a second researcher (CJ) analysed them for their key concepts. Focused coding was conducted using constant comparative methods [30, 31] with codes mapped using a preliminary causal-consequence model that identified the situational factors, conditions (enablers and barriers), strategies and outcomes of workforce development [30, 32]. This process resulted in our preliminary hypothesising about pathways underlying processes and systems to strengthen the workforce.

To test our preliminary theoretical perspectives and get a broader range of perspectives, interviews were then conducted with a wider range of Gurriny staff who expressed interest in participating in the project. Repeating the grounded theory analyses described above resulted in identification of key concepts regarding the strategies to strengthen Gurriny’s workforce. In total, interviews were conducted with 17 Gurriny staff members from various organisational positions, including senior and middle managers, as well as front-line workers from the clinical, health promotion and social and emotional wellbeing teams. Staff interviews included local Indigenous staff and non-Indigenous staff members. All interviews were conducted using an interview guide developed for this study (see Additional file 1). Findings were presented to the Gurriny Senior Management Team and staff, with their feedback incorporated into this analysis. The complexity of the Indigenous PHC context and importance of detail in doing justice to the resultant robust conceptual framework led to our decision to focus in this paper primarily on the strategies identified for informing Indigenous PHC models of workforce development. Further details of the analysis are available at https://www.lowitja.org.au/page/research/research-categories/health-services-and-workforce/workforce/completed-projects/working-well [33]. Given the relatively small (and potentially identifiable) number of staff participants, no participant descriptors are given for the quotes presented in this paper.

## Results

Analyses of interview data revealed a range of supportive workforce strategies that were already being implemented, and strategies for additional potential workforce improvements at the study site. We also briefly outline the enabling conditions which supported the implementation of these strategies.

### Strategies

**Fig. 1:**
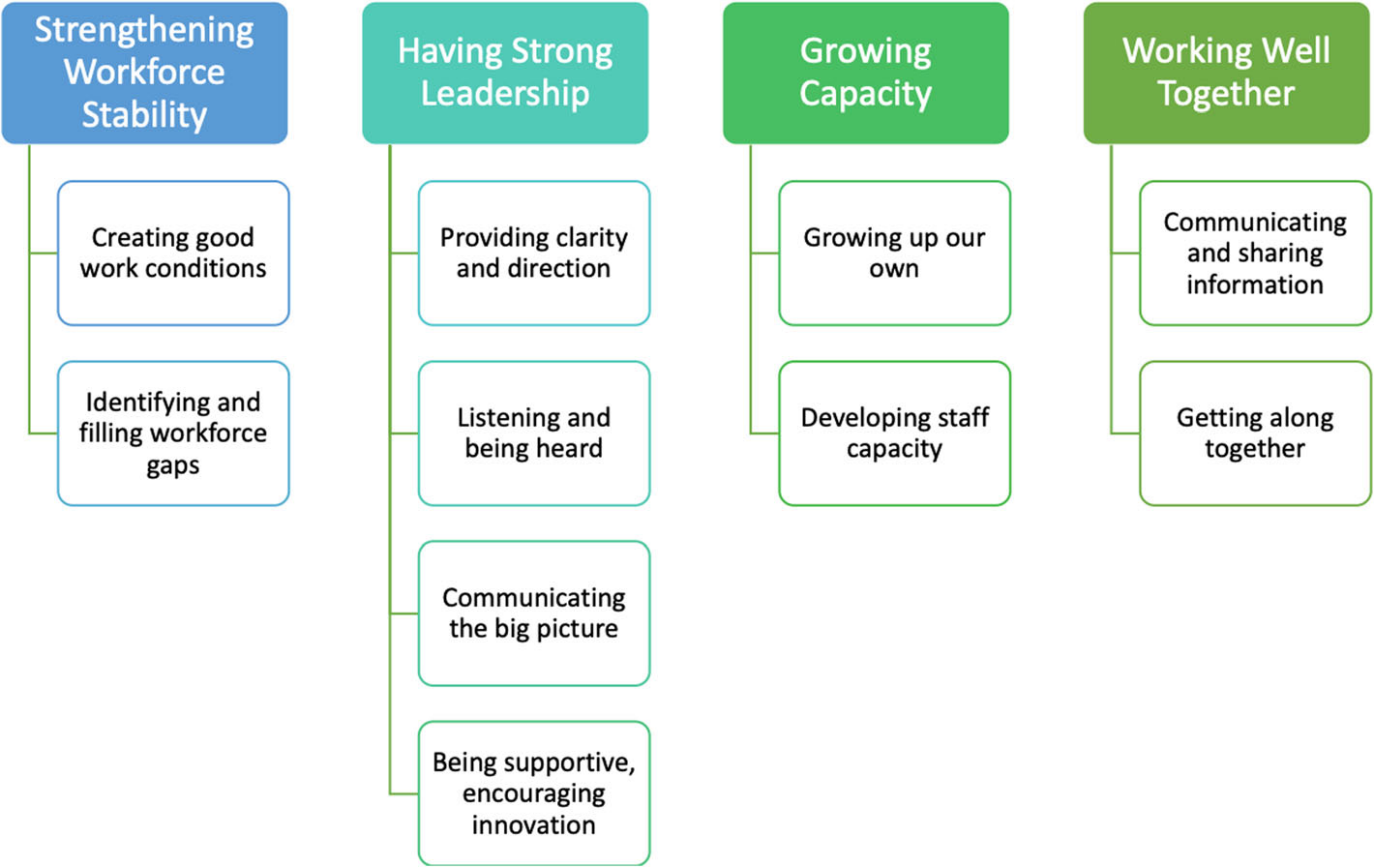
Model for workforce enhancement in IPHC

All identified strategies fall under the overarching categories of *Strengthening Workforce Stability, Having Strong Leadership, Growing Capacity,* and *Working Well Together.* These strategies can be built on into the future to help maintain and strengthen the workforce (Fig. 1).

#### Strengthening workforce stability

Whilst the ACCHS had a stable General Practitioner (GP), IHW and senior management workforce, it experienced a high turnover among nursing staff, and a need for additional male IHWs. To address nurse workforce turnover, participants suggested improved Continuing Professional Development (CDP) planning and opportunities and appropriate leadership for nurses.

*“one of the difficulties over that time is being able to establish enough of an RN* (registered nurse) *support workforce … that’s probably the area where there’s been more attrition.”*

To achieve workforce stability, participants also cited a need for staff to understand and agree with the values and philosophies of community control and comprehensive primary health care (PHC) and to have the right skills and experience.

*“I think that we’ve gotta be really careful for what we shop for … because I think we’ve for too long put bums on seats and that’s cost us greatly … I think that that person needs to be infused with our way … and really get the philosophy of community control”*

Stability in the workforce was supported through the work conditions provided to staff. Positive work conditions included generous flexibility in roles and responsibilities, study support and training opportunities, leave conditions, and work schedules to account for changes in personal circumstances.

*“I undertook my diploma … I done it here based in Yarrabah … luckily Gurriny has … Thursday afternoons* (for professional training and professional development) *and luckily my training fell on Thursdays so it just worked out well over that year”*

Staff also identified supportive benefits such as extra paid leave over the Christmas break, more paid study leave than industry standards, additional paid funeral leave and a paid day off on birthdays.

*“even though you get so sick, you use up all your sick leave, you use up all your holidays, they still support you”*

Participants suggested improvements to work conditions such as assuring that pay rates were consistent with roles, responsibilities and award rates, providing annual salary increases, and reconsidering the award wages for Health Workers and Nurses.

*“a lot of them are still sitting on the same wages as before”*

Also suggested were strategies to further enhance work conditions such as through ensuring that all staff had support in their daily responsibilities from a team or other staff member; increasing the regularity of Rostered Days Off (RDO’s), defined as an additional day in a roster period that an employee does not have to work [34], to once a fortnight as previously allowed, to support staff wellbeing, motivation and energy levels; and sharing responsibilities among staff to reduce work pressure.

*“R-D-O’s, we have it once a month and I think that has kind of impacted on some of our staff. Myself, I am starting to get really tired … at least when we had (RDO’s) once a fortnight, you know you had time.”*

While improvements in work conditions was an important theme, a number of participants identified a lack of awareness among some staff members of the relatively positive work conditions provided by Gurriny in comparison with conditions frequently provided in the health care sector. This suggested a need for greater understanding and appreciation of comparative conditions in the sector.

*“I don’t think the workforce actually … appreciate how lucky they are. The … conditions that they have, that they’re offered here are out of this world compared to mainstream.”*

#### Having strong leadership

Strong leadership is essential for a strong and stable workforce. Strong leadership was demonstrated at the service through clarity and direction provided by stable leaders. Strategic vision and direction has steered the service forward via understanding of the broader Indigenous PHC funding and policy contexts. The service has also had stable clinical leadership with an understanding of the needs and challenges of the medical workforce and has provided the support and guidance needed.

***“****the Senior Medical Officer role is not just to be in clinical governance, but is absolutely supporting that medical workforce and putting all the enablers around that so that they can practice efficiently … to mentor and supervise the group of doctors”*

The clarity and direction of organisational leadership could be strengthened by creating profession-specific leadership positions for both nurses and IHWs to ensure these roles are supported by leaders with the appropriate knowledge, skills and experience.

*“just like we have a Senior Medical Officer for the doctors, I think the nurses need like a Nursing Manager or a Team Leader”*

More clearly distinguished lines of leadership and greater clarity in leadership responsibilities and role boundaries could be achieved by creating team leader positions to simplify chains of command and address the issue of managers’ managing too large or multiple teams. Also, participants cited a need to clearly define the boundaries of senior management roles and responsibilities.

*“some roles go outside of where they should be sitting as well. The S-M-T (Senior Management Team) should be more defined in their responsibilities.”*

Strategies supporting strong leadership included senior managers have an open-door policy and middle managers being very responsive. There is also a formal system for responding to staff conflicts. However, participants suggested that further formal processes and opportunities could be created, and greater efforts put into creating a workforce culture that encouraged staff input into decisions and strategic direction.

*"there’s lots of great things going on, but there’s no consultation. Like with the whole organisation, to be a great leader you’ve gotta include your staff in decision making and feedback”*

Strong leadership is also about bringing staff along on the journey of organisational growth and improvement by communicating about and including staff in the strategic direction and decision making of the organisation. This is supported through the organisations operational management forum, which is designed to include operational managers in the broader strategic direction so they can better understand and share that understanding with their teams.

*“the middle managers … they’re able to be a part of understanding the strategic direction of the Board, filtered down to them from the Senior Management Team … then at least could share back with their team members, where we’re heading and why some of the decisions were being made, what directions were happening.”*

Participants suggested that leaders could support staff to better understand the broader Indigenous PHC and ACCHS sector contexts, as well as policy, funding and regulation contexts and how these impact on and limit what an individual service can do. For instance, some staff expressed a tension between the drive for local leadership and the need for professionals with the knowledge, skills and experience to understand that broader context. Staff members’ understanding of comprehensive, holistic primary health care and the role of Social and Emotional Wellbeing services in this model of care could also be strengthened.

*“my understanding was okay, well it’s community-controlled, community-driven, a hundred per cent local staff. But no … so when community heard about Gurriny’s going into community control, they didn’t make it clear what is community control”*

Finally, participants said that leadership is supportive by being available and approachable, providing encouragement and positive feedback to staff, and having rewards and incentives.

*"whoever worked well … that team will get a certificate for working well… so that inspires us to keep up the good work … That sort of really boost us up and help us”*

Leaders also encourage innovation and flexibility among staff by encouraging change positivity as well as promoting creativity and innovation in program design and delivery.

*“the beauty of working here was the creativity. They said basically, this is it. You can go for it… we could be free to reinvent what Queensland Health had just said, ‘this is how your care’s going to look.’”*

However, leaders could do more to encourage staff members to share and implement ideas and solutions, and to provide encouragement and positive feedback in both formal and informal ways to ensure that staff feel valued.

*“I think that we can then sit down and say, ‘well how about let’s jump out of the box. Let’s just take this to a whole new level.’”*

A member of the senior management team reflected at the research feedback session that Gurriny’s leaders could do more to enhance communication mechanisms with staff. Suggestions included an open email circulation of the findings of the research, and an active notice board for two-way communication of issues and suggestions.

#### Growing capability

To strengthen the workforce there is a need to build the capability and capacity of staff. Strategies already being implemented to grow up the local health workforce are focused on providing opportunities to build the knowledge, skills, experience and capacity of local staff. These strategies include: supporting IHWs to gain qualifications; providing scholarships to support professional development and paid study leave; allowing trainee entry points; supporting staff to present at and attend conferences nationally and internationally; providing mentorship and staff education; and supporting leadership building opportunities.

*"I think Gurriny is very big on professional development … Thursday’s we have professional development day, so there’s always something happening for us around professional development."*

However, a strong desire was expressed by participants for further strategies to support strengthened local leadership, in particular, to increase the number of local Indigenous people in senior management positions. Suggestions included career progression pathways for local staff by creating more middle management and team leader positions, giving local staff a chance to move into leadership positions, and increasing wages with increased qualifications, experience and responsibility. Recognising and rewarding staff effort, determination and ambition is important to raise up the next generation of leaders. Also, strong succession planning was an identified need, including clear career progression pathways and systematic processes for staff to follow.

“*We’ve been asking, ‘where’s the succession plan for this organisation?’ … you’ve got young ones there that are energetic. That want to be in that leadership position, and they can do it as well. You know, these are local people … they should’ve identified … what skill-sets do they have? Are they able to work at that level now or how can we build their capacity say in the next five years?”*

Local leadership could also be strengthened through enhancing health worker capacity. Suggested strategies included improving health worker training by situating the skills needed in their broader context and providing opportunities to apply the knowledge and skills learnt in training, or by supporting IHWs to become Health Practitioners. Finally, participants identified a need for greater clarity about the role of non-Indigenous staff in supporting local staff career progression and leadership capacity development. This could be achieved by establishing mentoring/role shadowing systems and building these into contracts and key performance indicators (KPI’s).

*"that’s why I sort of always advocate and say you know when we’re doing health checks and assessments, let the Health Workers do it to build their clinical skills up too. Because if they don’t, they’re gonna lose it"*

Support for general development of staff capacity, both local Indigenous and non-Indigenous, is provided through having accessible in house and external training and education opportunities in Yarrabah and Cairns as well as paid or unpaid study leave; the creation of middle management positions to support career progression pathways; and developing senior and operational management skills and capacity.

*“So these training were offered in Yarrabah which was made accessible and easy to attend”*

Strategies to better support the development of staff capacity included ensuring that the approval of training and professional development requests were fair and equal and supporting nurses to maintain clinical skills and professional requirements through sufficient CDP planning and activities.

*“I feel that my skill set has definitely deteriorated since I’ve been here.”*

#### Working well together

Finally, a strong and stable workforce requires strategies that support a positive work culture where staff members work well together, and feel good in the workforce. Staff members were supported to work well together through clear communication and information sharing systems and processes such as regular team and all of staff meetings, having team plans with matching KPI’s, effective email communication, and utilising the Operational Management Forum to communicate and share information across the organisation.

*“each Monday, we come to work, eight o’clock we have our meeting with everyone, all Health Workers, all doctors, all nurses … and then when we come away, then the doctors and the nurses stay behind for their meeting. And our team- the Care Coordination team, we have our meeting down at the back … then at least we will know what each one is doing”*

However, there is a need for improved information sharing between staff, teams and management about services, work conditions and benefits, others’ roles and responsibilities, and professional development and training opportunities. Suggested strategies included the provision of clear guidelines and instructions; longer, more in-depth induction training to teach the ACCHS philosophy and model of care; and clarifying staff expectations around roles and responsibilities as well as benefits and entitlements, such as training, leave and registered days off. These strategies could be facilitated by increasing transparency from senior and operational management and having regular meetings with an open agenda focused on giving staff the opportunity to raise and discuss workplace issues. A staff member present at the research feedback session also suggested that staff understandings of the philosophy of community control could also be usefully enhanced by incorporating within the staff induction process a history of what motivated Gurriny’s transition to community control, the process entailed in that transition, and the outcomes.

*“our induction processes … I think it’s over a day. And I think it just needs to be over a week and I think that that person needs to be infused with our way … and really get the philosophy of community control”*

Staff work well together when there is cohesive team culture in which people get along. Participants noted that Gurriny staff are there for one another, help each other out, are kind to one another and are understanding of personal context. They get past personal differences and maintain respect and professionalism. In multidisciplinary teams, staff from all different positions and programs, Indigenous and non-Indigenous, share knowledge, skills and experience. The ACCHS supports this process through creating opportunities for cross-organisational bonding, including formal and informal whole of organisation and team/program specific team building events.

*“I really think that everyone has respect for one another. I think everyone, they do well to come to work and put their differences aside. Everyone always works towards positive outcomes, regardless of whatever issue”*

The ACCHS workforce could be further supported to work well together by clarifying expectations and understandings of the impacts of personal circumstances, as well as creating processes to work through and overcome personal differences. There could also be improvements in encouraging and facilitating staff members to work outside of role and program boundaries and to better integrate clinical and social and emotional wellbeing (SEWB) programs and services, as well as efforts to create further opportunities for cross-organisational bonding.

*“you’ve gotta kind of have a sense of … how other people operate in your space. When you assume that a person maybe is not doing their job properly or is undermining you, it’s not at all, it’s just that they’re coming from it from a completely different angle.”*

### Enablers

Enabling conditions were also identified for implementing the four strategies 1) Strengthening Workforce Stability; 2) Having Strong Leadership; 3) Growing Capacity; and 4) Working Well Together. Briefly, the key enabling conditions for developing a strong ACCHS workforce occurred at macro, community, organisational and individual levels. Macro-level enabling conditions include national and international movements and discourses of Indigenous self-determination and community control as well as government commitment and support for the transition to community control. Enabling conditions at the community level include local ownership and control of PHC service and social and cultural dynamics, norms and systems.

*“at the Senior Management level … (Gurriny has) six and three of them are Indigenous. Plus you’ve got your Board as well. And then the Operational Management level, at this point in time, every Manager is Indigenous.”*

At the organisational level, enabling conditions include Gurriny’s process of organisational growth from before, during and after transition to community control; strong local governance; holistic, comprehensive trauma-informed and family-centered model of care; flexibility in service delivery; as well as increases in senior and middle management positions to support organisational growth.

*“our model of care, we’re looking at it being culturally appropriate. We’re looking at it being a preventative right through to acute. It’s counselling services, it’s Mental Health, it’s Child Maternal health etcetera, but also, we’re trying to broaden out the model of care to describe how the interaction between the staff operates”*

Finally, on the individual level, enabling conditions included strong professional boundaries, change positivity, staff motivation and commitment, strong local leadership and a large percentage of local, Indigenous staff who are motivated by a strong responsibility to improve the health and wellbeing of the Yarrabah community.

*“It’s not just about being paid … it’s about these people. The rest of our mob out there. We have close family members that we need to help”.*

Enablers at all four levels were required to optimise implementation of the strategies outlined above.

## Discussion

The strategies to strengthen the Indigenous PHC workforce found in this study reflect many of those established in the literature [8–11]. *Strengthening Workforce Stability, Having Strong Leadership, Growing Capacity,* and *Working Well Together* are broadly similar to the three multifaceted strategies found in our recent literature review: 1) enhancing recruitment and retention; 2) improving supervision, mentoring and support; and 3) strengthening roles, capacity and teamwork [11]. They are also supported by the six priority areas of the National Aboriginal and Torres Strait Islander Health Workforce Strategic Framework 2016–2023 [6]. In addition, this study identified several novel strategies to strengthen the Indigenous PHC workforce including ensuring profession specific leadership and providing opportunities for Indigenous PHC staff to have input into decision making. Workforce needs and challenges unique to the ACCHS sector are also discussed. This study outlined many positive and innovative strategies being implemented in Indigenous PHC to support staff professional development. It also reiterates the identified need for further efforts to build IHW capacity across these key areas.

Workforce stability is closely associated with workforce conditions. Inadequate remuneration is a systemic issue in Indigenous PHC where low salaries result from low, short-term and non-recurrent funding, especially in the non-government sector [8]. Remuneration levels are a sensitive indicator of employee mobility in rural and remote health in general, therefore maintaining realistic and competitive remuneration is an important retention strategy [10]. Adequate remuneration is a major issue particularly for IHWs who are more likely to have lower wages than other occupational groups [8], with pay inequality noted as one of IHWs major frustrations [35]. Suggestions to address this issue include IHWs organising together across Australia to increase award wages as well as organisations reviewing staff salaries and negotiating with funding bodies to ensure budgets provide appropriate remuneration [8]. Other important strategies to support the stability of the Indigenous health workforce include culturally appropriate human resources management practices such as adequate leave provision for cultural commitments and those which support professional development such as subsidising study costs or allocating work time to study purposes [8].

Strong leadership, expressed through clear communication and strong management and supervision, has also been identified as important for fostering effective and sustainable workplaces in rural and remote healthcare [10]. Research on factors affecting workplace motivation for health and human services professionals has identified the importance of honest, open, appropriate and timely communication; a sense of being respected and valued; positive, regular, timely and specific feedback; and a clear direction from leadership [36, 37]. Indigenous PHC leaders need to find ways to effectively communicate with staff about complex issues related to the Indigenous PHC and ACCHS sectors as well as ensure transparency and consistent and effective information sharing.

The importance of profession-specific leadership was a novel finding of this study. A lack of such leadership which understands the professional context, needs, experience and skills of groups of Indigenous PHC professionals can leave staff feeling unsupported in their roles and in the organisation. This study also emphasised the significance of Indigenous PHC leaders consulting staff and providing opportunities to have input into decision making. Involvement in decision making, has been shown to increase organisational commitment [38]. This may be of particular importance for local staff who feel a sense of ownership in the ACCHS context which prioritises Indigenous self-determination.

Professional development opportunities in the form of training and education are important for growing the Indigenous PHC workforce capacity by developing both clinical and non-clinical skills and competencies [8, 11, 39]. Innovative implementation of key strategies to support the development of staff capacity demonstrated in this study include the provision of on-the-job training and education through the employment of staff educators and providing internal in-service courses and training through a full afternoon dedicated to professional development weekly. Other studies have found that it is important that Indigenous PHC services support diverse training pathways by providing opportunities for both formal education and training as well as the more informal mentoring and work shadowing which may be more appropriate for Indigenous practitioners [11]. Furthermore, professional development opportunities should be tailored to the need of the local health workforce [39], supporting the development of specific skills and knowledge related to health programs and the needs of the intended patient group [9], while also being relevant to career advancement for health professionals [11]. This can be achieved through CPD plans which clearly establish professional development needs and goals [8].

Professional development opportunities for IHWs may have improved over the last few decades [35], however, this study identified ongoing issues such as IHWs not having opportunities to implement new learning in the workplace after the completion of training [8, 35] and feeling underutilised in the workplace [8]. Strategies to address these issues include enhancing IHW roles, providing greater opportunities to apply skills in practice, improving IHW training, and ensuring that IHWs have opportunities to work to their full scope of practice [11, 39]. This is complicated by the substantial variation that exists in IHW roles, definitions, scopes of practice, education standards and career pathways [14]. There is a need for clearer definition of roles and enhanced role recognition among IHWs to better support their professional development and career progression [11, 14].

Another important area for Indigenous PHC workforce enhancement, particularly in ACCHSs, is in relation to career progression pathways for local staff [8, 11, 39]. In rural and remote healthcare in general, opportunity for career advancement is an important catalyst for retention [10]. The frustration experienced by IHWs in regard to lack of opportunities for career progression in both government and ACCHS sectors is well established [35], with such limited career pathways causing some IHWs to look for work outside the health sector [8]. This issue can be addressed through the development and implementation of succession plans and strong, clear career pathways [8, 11, 39]. Providing recognition for increased qualifications by changing role descriptions and providing appropriate remuneration are also important strategies [8].

In addition to strengthened career progression pathways for Indigenous staff, there is an identified need to provide opportunities for the development of leadership capability among the Indigenous workforce at all levels; from entry to leadership positions [11, 39]. This study identified a strong sentiment in ACCHSs regarding the need to have more locals in leadership positions, particularly in senior management. One strategy to support this is mentoring. Yet, despite the recognised importance of mentoring [8, 39] there is a limited literature documenting formal mentoring strategies in Indigenous PHC [11]. Strategies to support local career progression and leadership capacity development at Gurriny were in the pipeline during this study, such as creating team leader positions. Overtime, the implementation of such initiatives could see a positive impact on this issue.

Working well together through a positive work environment, with friendly people and strong team was the final strategy to support a strong workforce [36, 37]. Informal structures to support team work, such as debriefs and meetings, as well as strong team cohesion have been identified as critical factors in building a strong Indigenous PHC workforce [11]. Mentoring and support from colleagues are particularly important for retention among Indigenous health professionals [8]. Successful efforts to create a strong and cohesive work culture demonstrated in this study include those targeting the whole organisation through all in staff meetings and bonding days, as well as those to improve communication and cohesion targeting senior and operational management.

Literature on human resources management (HRM) perspectives can contribute to our understanding on workforce challenges facing Indigenous PHC [40]. For example, HRM literature on psychological contracts offers a useful perspective to help understand why staff in Indigenous PHC services, and ACCHSs in particular, may feel frustrated or disappointed by the paucity of career progression and leadership opportunities for local staff. A psychological contract can be defined as “a set of individual beliefs or perceptions regarding reciprocal obligation between the employee and the organisation” (p. 57) [41]. While some of these are formally recorded in a written contract, they are mostly implied and not openly discussed. Psychological contract violation occurs when there is a perceived failure to fulfil obligations or promises in the workplace [41]. Such violation can lead to feelings of anger, distress, betrayal, resentment and a sense of injustice, which in turn can lead to job dissatisfaction and reduced organisational commitment, as well as negative impacts on role performance, and increased staff turnover [36, 41]. The concept of psychological contract violation is a potentially powerful explanatory theory which can help to understand several of the workforce issues raised in this study, including: professional development opportunities; career progression; pay rates; and the ability to include decision making. For example, as one important area of potential psychological contract violation, participants suggested an expectation by some local staff that they would be promoted to leadership positions within the ACCHS [41].

To overcome such violations, it is important that the perceptions of obligations and promises are shared between management and employees [41]. There needs to be clear and honest communication of expectations and obligations of the employee, as well as the organisation, starting from recruitment and orientation. Regular performance reviews provide opportunities to dispel false beliefs regarding expectations as well as to agree upon future strategies for professional development [41]. In the ACCHS context there needs to be clarity from the outset about what the organisation can do and what staff will need to do in order to progress to leadership roles, ie. minimum education or skill requirements for senior management roles. Further research into psychological contracts and psychological contract violations among Indigenous Health Professionals in Indigenous PHC, particularly for ACCHSs in discreet Aboriginal communities where there is an expectation of local leadership is needed.

The findings of this study largely reflect what is identified in the established literature on the workforce strengths and challenges faced by Indigenous PHC [8–11] and therefore can be of value to other Indigenous PHC services looking to strengthen their workforce. They also provide insight into workforce needs and issues which may be unique and/or be of particular importance in ACCHS contexts. Further research into the workforce experiences and needs of ACCHSs in varied organisational and community contexts is required to understand if the findings of this study are relevant to other ACCHSs. Finally, considering that there are many similarities between the workforce needs and challenges of Indigenous PHC services and mainstream PHC services [10] the findings of this study may be relevant to non-Indigenous PHC services providing healthcare to Indigenous Australians.

### Limitations

While the findings of this study may hold relevance for other Indigenous PHC services, ACCHSs and the broader Australian PHC service sector, this study was undertaken with only one ACCHS therefore caution should be exercised when applying these findings to other contexts. Considering the important differences that exist in geographical, community and organisational contexts between different Indigenous PHC services, the results of this study may need to be tailored for relevance to other ACCHSs/IPHC services. Only 17 of Gurriny’s 76 staff were interviewed; however, they were representative of the diversity of the workforce Indigeneity, professions and roles. We acknowledge that there can never be complete analysis of data or that our explanatory theory or conceptual models can ever be absolute [42] but consider that the rich data provided has led to a conceptually robust identification of the core strategies that support a strong Indigenous PHC workforce development.

## Conclusion

This paper outlines a range of promising strategies which can be used in workforce enhancement efforts for Indigenous PHC. When taken together, these strategies provide a comprehensive set of recommendations, informed directly from ACCHS staff, about pragmatic strategies for strengthening the workforce culture of Indigenous PHC services and creating the type of stable, capable and cohesive workforce that is needed to be able to effectively meet the health needs of Aboriginal and Torres Strait Islander populations. Health workforce issues are complex and multifaceted, with no clear one-size-fits-all approach that is going to be effective everywhere [10, 43, 44]. The workforce challenges of each organisation will be different, therefore decisions about particular strategies should be tailored to local needs and issues [10]. While confirming much that has already been outlined in the literature on Indigenous PHC workforce strengths and challenges, the current paper raises some important issues and questions regarding workforce issues specific to ACCHSs, which may or may not be different depending on whether the ACCHS is situated in a discrete Aboriginal community. There is a need to further investigate these identified issues and for sound evaluation of workforce improvement strategies throughout implementation to build the evidence base.

## Supplementary information

**Additional file 1.** Interview guide. The interview guide is a list of primary and secondary probing questions which were used in the interviews with participants for this study.

## Data Availability

The datasets generated and analysed during the current study are not publicly available due to participant confidentiality but are available from the corresponding author on reasonable request.

## Abbreviations

ACCHS: Aboriginal Community Controlled Health Service
ATSIHW: Aboriginal and Torres Strait Islander Health Workers
CDP: Continuing professional development
GP: General practitioner
HRM: Human resources management
IHW: Indigenous health worker
KPI: Key performance indicator
NACCHO: National Aboriginal Community Controlled Health Organisation
PHC: Primary healthcare
QH: Queensland Health
RDO: Rostered day off
RN: Registered nurse
SEWB: Social and emotional wellbeing
SMT: Senior management team
