# Supplementary material for: Working well: strategies to strengthen the workforce of the Indigenous primary healthcare sector
Source: BMC Health Serv Res. 2019 Nov 29;19:910. doi: 10.1186/s12913-019-4750-5 (PMC6883573; doi:10.1186/s12913-019-4750-5)
Supplement: Supplementary file 1 — Additional file 1. Interview guide. The interview guide is a list of primary and secondary probing questions which were used in the interviews with participants for this study. [file 12913_2019_4750_MOESM1_ESM.docx]

**Working Well: Tailoring a workforce development model to deliver sustained improvements in community controlled healthcare.**

**RESEARCH QUESTION:** *What is working in Gurriny to support staff capacity, leadership, career development, motivation and wellbeing, what is not working, and how can this be improved?*'

**Interview questions**

CAPACITY BUILDING and CAREER DEVELOPMENT

Can you tell me about your position here at Gurriny? What team are you part of?

How long have you been in your current position?

Is your role and what’s expected of you clear?

Can you tell me about how the XXXX team works together, and if you have any thoughts about how it could be improved?

*Do you feel supported by the team?*

*Do you have the resources you need for the team to work well?*

If Gurriny was to create new positions, what professions and skills do you think are needed most?

Would you like to progress in your career at Gurriny?

Do you feel like you have opportunities to progress in your career?

*What are your career goals?*

*Are there things you feel might help you achieve your goals?*

*Are there things that you think might stop you from achieving your goals?*

Are you satisfied with your opportunities for professional development?

*What professional development have you attended in the past year?*

*Do you feel supported by Gurriny in your professional development?*

*In what ways does Gurriny support professional development?*

Have you had experiences of being mentored, or providing mentoring for others?

If so, what was that experience like? What did you gain? How did it help you in your job?

**LEADERSHIP**

Do you think there is enough Indigenous leadership at Gurriny?

*If so, how is this achieved?*

*If not, how do you think Indigenous leadership at Gurriny could be improved?*

How do you think the leadership role of Aboriginal Health Workers could be improved?

What is your understanding of the role of the Gurriny Yealmucka Board of Directors?

*Do you think the board provides effective local leadership for Gurriny? If so how? If not, why?*

What is your understanding of the role of the Members of the health service corporation?

*Do you think community membership provides effective local leadership for Gurriny? If so how? If not, why?*

*What would be a good way to get feedback from patients and the community about the service?*

Do you feel that you are well informed by the Gurriny Senior Management Team about what is happening in Gurriny?

Do you have suggestions for how communication channels could be improved?

**MOTIVATION**

Do you think that Gurriny workers feel motivated to come to work and do their work?

*Why would staff not be motivated?*

*Why do you think it’s an issue?*

*Why do staff struggle with motivation?*

Do you feel like you understand the roles of others at Gurriny?

Are you interested in learning more about the roles of others?

Would you like to learn enough so that you could do some of the work that other people do in their roles?

How well do you think staff members get along with each other at Gurriny?

Can you give any examples of what Gurriny does well to support staff to get along together? And/or what they could do better?

Have you had (or know about) experiences of staff conflicts or disagreements/tensions from disagreements?

How was it dealt with and do you think it got sorted out?

How could it have been better dealt with?

**PROFICIENCY**

Do you feel you have the skills and experience needed to address the healthcare needs of the Yarrabah community and perform well in your role?

(Non-local staff members only) How do you get feedback on whether you’re doing the right thing culturally in the care that you provide?

is there a better way of getting this kind of feedback?

**WELLBEING**

Do you think that Indigenous staff members have extra responsibilities (or stressors) in their work compared to other staff because of expectations from community members (and living conditions or health issues)?

If yes, can you describe these responsibilities (and/or stressors)?

What are the effects of these responsibilities (and/or stressors) on the way you/they are able to do your/their job?

How do you/they manage these responsibilities (and/or stressors) to take care of your/themselves?

How do you think Gurriny could better support the wellbeing of local employees?

What do you think are the stressors for non-local staff in working at Gurriny?

How do you think Gurriny could better support the sense of belonging and wellbeing of non-local staff?

Do you think staff turnover is an issue at Gurriny? If so, where do you think the majority of staff turnover occurs?

What things support staff to feel good and stay in their position?

What things make it difficult or doesn’t support staff to feel good and stay in their position?

What are some of the things that make Gurriny a good place to work?

What are some things that could make Gurriny a better place to work?

What are the best things about your job?

What things are important to make you want to stay in your job?

What are the hardest parts of your job?

Can you think of things that would make your job more satisfying?
